# Diversity and Functional Prediction of Gut Microbiota in Forficulidae Natural Enemies from Mulberry Orchards and Cornfields in Southern China

**DOI:** 10.3390/insects17050512

**Published:** 2026-05-18

**Authors:** Yanli Zheng, Qiwen Yan, Qiwei Chen, Guangjie Luo, Yan Yang, Xuejian Wang, Shuang Yang, Dandan Liu

**Affiliations:** 1 School of Geography and Resources, Guizhou Education University, Guiyang 550018, China; yanli929718@163.com (Y.Z.); 2522050001@gznc.edu.cn (Q.Y.);; 2 Guizhou Provincial Key Laboratory of Geographic State Monitoring of Watershed, Guizhou Education University, Guiyang 550018, China; 3 Mountain Research Institute, Guizhou Education University, Guiyang 550018, China

**Keywords:** Dermaptera, high-throughput sequencing, different host plants, midgut bacteria, different geographical regions, development and utilization

## Abstract

To clarify the compositional characteristics and functional mechanisms of gut microbial communities in wild earwigs (Dermaptera) and explore the potential of earwigs for development and utilization as natural enemies, this study conducted a comparative analysis of the gut microbial diversity and community structure of two earwig species, *Timomenus komarovi* (Semenov, 1901) and *Eudohrnia metallica* (Dohrn, 1865), which are widely distributed in mulberry orchards and cornfields of Guizhou Province, China. It also predicted the microbial functions based on the third-generation high-throughput sequencing technology targeting the 16S rRNA gene. The results showed that the two earwig species across different habitats and geographical regions harbored a similar core microbial flora. The dominant phyla of gut microbiota were Proteobacteria, Firmicutes. PICRUSt2 functional prediction analysis revealed that the functions of intestinal bacteria in earwigs were mainly concentrated in metabolism-related pathways. Through multi-dimensional analysis, it was confirmed that the gut microbial communities of earwigs were constructed following the “core-specialization” model. The core microbial communities exhibited high conservatism. Host species were the core factor shaping the composition of earwig gut microbial communities. Habitats could further regulate the commonness and diversity characteristics of the microbial communities, while geographical regions had an extremely weak impact on the gut microbial communities. Significant differences were observed in the gut microbial adaptation strategies between the two earwig species, which determined their distinct development potential and application scenarios as natural enemy insects: *T. komarovi* is suitable for development as a broad-spectrum natural enemy, while *E. metallica* is more appropriate for targeted development based on its microbial community characteristics. All data supporting the findings of this study are accessible in the NCBI database under BioProject accession number PRJNA1449822. This study provides a theoretical basis for exploring the functional mechanisms of intestinal microbes in dermapteran insects and supporting the development and utilization of these natural enemy resources.

## Simple Summary

To explore the potential of earwigs (Dermaptera) for development and utilization as natural enemies, this study selected two earwig species widely distributed in mulberry orchards and cornfields of Guizhou Province in China as research objects. High-throughput sequencing technology was applied to conduct an in-depth investigation about the gut microbiota of earwigs across different geographical regions, habitats, and species, revealing the community composition, diversity characteristics, and potential functions of the microbiota. These findings clarify the compositional features and functional mechanisms of gut microbial communities in wild earwigs, which lays an important foundation for understanding the host–microbe interaction mechanisms of this group and provides a theoretical basis for the conservation and utilization of wild natural enemy insect resources.

## 1. Introduction

Earwigs (Dermaptera) demonstrate an extremely high level of environmental adaptability and play a dual role as predators and herbivores in ecological systems, making them important natural enemies of agricultural and forestry pests [1]. Numerous studies have verified their outstanding predatory ability [2,3,4,5,6,7,8], they can effectively prey on a variety of agricultural and forestry pests, such as *Ostrinia furnacalis* (Guenée, 1854), *Psylla* spp., *Aphis* spp., and Thysanoptera spp. in orchards and farmlands, thereby providing valuable natural enemy resources for green pest control in agriculture.

Insect gut microbiota participate in almost all physiological processes of the host, including feeding preference, nutrient utilization, lifespan regulation, immune response, insecticide resistance, and environmental adaptability [9,10,11,12,13,14,15]. The diversity and richness of gut microbiota are regulated by multiple factors, among which host species, genotype, food source, and living environment are the key influencing factors [14,15]. For predatory natural enemy insects, gut microbiota play a central role in maintaining host health, regulating nutrient metabolism, and enhancing environmental adaptability, and their community composition is jointly shaped by host species, food sources, and environmental factors [16,17,18]. Diet, as a key environmental factor, significantly drives the variation in the composition and diversity of insect gut microbiota [19,20,21,22,23]. During the adaptation to different food resources, the host can counteract the adverse effects caused by dietary changes through the regulation of gut microbial community structure and further form a specific gut microbiota that matches its own feeding habits [22,23]. Polyphagous insects, such as *Spodoptera frugiperda* [9,24,25,26,27], *Drosophila melanogaster*, and *Grapholita molesta* [28,29], show significant differences in the richness and evenness of gut microbiota when feeding on different host plants. Secondary metabolites of host plants further filter microbial communities [30], suggesting that diet regulates microbial niches through a bottom-up mechanism [27,31,32]. Nevertheless, the community composition and underlying functional mechanisms of the gut microbiota of omnivorous earwig natural enemies are still poorly understood.

**Figure 1:**
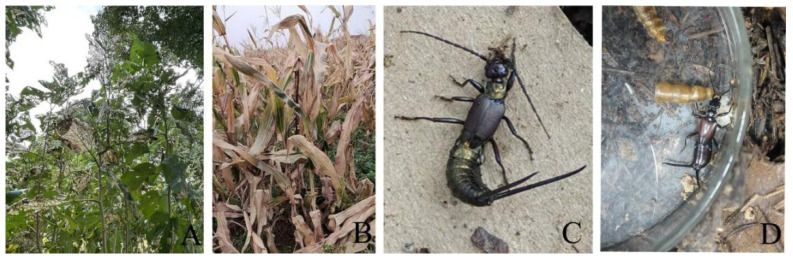
Earwig samples and their collection habitats. (**A**) Mulberry garden; (**B**) Cornfield; (**C**) *E. metallica* (Dohrn, 1865), female; (**D**) *T. komarovi* (Semenov, 1901), female.

To elucidate the composition and functional mechanisms of gut microbiota in wild predatory earwigs from Guizhou Province, China, we selected two native earwig species as research materials. The two species, *Timomenus komarovi* (Semenov, 1901) (Figure 1D) and *Eudohrnia metallica* (Dohrn, 1865) (Figure 1C), are widely distributed in local mulberry orchards and cornfields [26].

Samples from different geographical regions were collected to extract the 16S rRNA gene of gut microbiota. The composition, structure, and diversity of gut microbiota in earwigs from different species, habitats, and geographical regions were systematically analyzed, and the functions of gut microbiota were predicted. To explore the development and utilization potential of these two indigenous natural enemy insects, this study aims to provide a theoretical basis for the conservation, artificial breeding, and efficient field utilization of native natural enemy insect resources.

## 2. Materials and Methods

### 2.1. Collection of Earwig Samples

Earwigs were collected from five distinct habitats in Guizhou Province by the authors from 1–7 October 2025, the number of specimens collected in each sampling site was more than 20 individuals at the same place. Samples were grouped based on the different habitats of the two earwig species. The group information of the sampling sites is detailed as follows: Group X, *T. komarovi*, Mulberry Orchards, in Xinpu Sub-district, Honghuagang District, Zunyi City, Guizhou Province (107°02′ E, 27°44′ N, altitude: 886.50 m); Group F, *T. komarovi*, Cornfields, in Fenggang County, Zunyi City, Guizhou Province (107°47′ E, 28°01′ N, altitude: 798.20 m); Group P, *T. komarovi*, Cornfields, in Xinpu Sub-district, Honghuagang District, Zunyi City, Guizhou Province (107°02′ E, 27°44′ N, altitude: 886.50 m); Group W, *E. metallica*, Cornfields, in Xiuwen County, Guiyang City, Guizhou Province (106°31′ E, 26°56′ N, altitude: 1256.80 m); Group D, *E. metallica*, Mulberry Orchards, in Puding County, Anshun City, Guizhou Province (105°29′ E, 26°17′ N, altitude: 1312.60 m). Species identification of earwigs was completed by the first author, Yanli Zheng, according to Reference [33]. The earwig specimens collected at night were transported back to the laboratory, where gut microbial sampling was conducted immediately.

### 2.2. Sampling Method for Intestinal Contents

According to established methods for dissecting gut contents from various insect species [34,35], intestinal sample preparation was carried out under aseptic conditions. Earwigs were placed on ice for 5 min to induce anesthesia before dissection. The body surface was sterilized by wiping with 70% ethanol for 30 s. Each individual was held by the thorax using sterile forceps, immersed in a 0.25% sodium hypochlorite solution for 1 min, and then rinsed three times with sterile water. Subsequently, dissect and take out the complete midgut in a sterile and liquid-free Petri dish and immediately washed twice with a 0.9% sterile NaCl solution. The midgut was placed into a 1.5 mL microcentrifuge tube, 200 μL of sterile normal saline was added, and the tube was stored at −80 °C for later use.

### 2.3. DNA Extraction, Library Preparation, and High-Throughput Sequencing

Genomic DNA extraction was strictly carried out following the manufacturer’s instructions of the E.Z.N.A.^®^ Soil DNA Kit (Omega Bio-Tek, Norcross, GA, USA). The concentration of the extracted DNA was determined via electrophoresis. Using the extracted total DNA of gut microbiota as the template, specific primers were designed for the V3–V4 region of the 16S rRNA gene. The primer sequences referred to universal primers (forward primer 341F: CCTACGGGNGGCWGCAG; reverse primer 806R: GGACTACHVGGGTWTCTAAT), and the primers were synthesized by a professional biotechnology company. The PCR amplification reaction system and reaction conditions were as follows: PCR reaction system (25 μL): 12.5 μL of 2 × Taq PCR MasterMix, 1 μL of forward primer (10 μmol/L), 1 μL of reverse primer (10 μmol/L), 2 μL of DNA template, and 8.5 μL of sterile double-distilled water. The mixture was vortexed to mix well and centrifuged briefly (5000 r/min, 1 min) to concentrate the reaction system at the bottom of the tube. PCR reaction conditions: pre-denaturation at 95 °C for 5 min; followed by 30 cycles of denaturation at 95 °C for 30 s, annealing at 55 °C for 30 s, and extension at 72 °C for 45 s; final extension at 72 °C for 10 min; and storage at 4 °C to terminate the reaction. All PCR amplifications were conducted on an ABI GeneAmp^®^ 9700 PCR System (Applied Biosystems, Foster City, CA, USA). After successful PCR amplification, the PCR products were subjected to gel cutting, recovery, and purification using the Axygen^®^ AxyPrep DNA Gel Extraction Kit (Axygen Biosciences, Union City, CA, USA). The target bands of the PCR amplification products of all samples were of correct size and appropriate concentration, which could be used for subsequent experiments. With reference to the preliminary quantitative results of electrophoresis, the PCR products were accurately quantified using Qubit^®^ 3.0 (Thermo Fisher Scientific, Waltham, MA, USA), mixed in the corresponding proportion according to the sequencing volume requirement of each sample for library construction, and the on-machine sequencing was completed with the assistance of Shanghai Lingen Biotechnology Co., Ltd. using the PacBio Sequel IIe platform (Pacific Biosciences, Menlo Park, CA, USA).

This study involved five experimental groups, namely Groups X, F, P, W, and D, and employed the pooled sequencing method. From each experimental group, ten earwigs were selected, and their intestinal tissues were dissected separately to extract gut microbial DNA. After passing the purity and concentration detection, the gut microbial DNA of all individuals in the same group was uniformly mixed in equal volumes to form one pooled sample, which was used for subsequent PCR amplification, library construction, and high-throughput sequencing.

Four replicates were randomly selected from each group for sequencing, and the sample IDs were designated as follows:

- Group X: X-24-1, X-24-2, X-24-3, X-24-4;
- Group F: F-22-1, F-22-2, F-22-3, F-22-4;
- Group P: P-11-1, P-11-2, P-11-3, P-11-4;
- Group W: W-24-1, W-24-2, W-24-3, W-24-4;
- Group D: D-24-1, D-24-2, D-24-3, D-24-4.

All data supporting the findings of this study are accessible in the NCBI database under BioProject accession number PRJNA1449822.

### 2.4. Statistical and Bioinformatics Analysis

OTUs (Operational Taxonomic Units) are artificially unified labels assigned to specific taxonomic units (such as strains, genera, species, and groups) in phylogenetic and population genetics research to facilitate data analysis. To obtain information on the number of bacterial species and genera from sample sequencing results, sequence clustering is required. All sequences are divided into numerous groups according to their similarity, and each group corresponds to one OTU. To obtain the taxonomic information corresponding to each OTU, the uclust algorithm was used for taxonomic analysis of OTU representative sequences at a 97% similarity level. The community composition of each sample was statistically analyzed at multiple taxonomic levels, including domain, kingdom, phylum, class, order, family, genus, and species. The 16S rRNA reference database for bacteria and archaea was adopted. By default, the SILVA database [36] (Release 138.2, http://www.arb-silva.de, accessed on 8 May 2026) was used without additional designation. Taxonomic annotation was performed using RDP Classifier [37] (Version 2.2, http://sourceforge.net/projects/rdp-classifier/) (accessed on 28 March 2026)), with the confidence threshold set to 0.8 for SILVA annotation and 0.7 for RDP classification. According to the sample grouping information, OriginPro 2024 was used to perform inter-group statistical difference tests on the total relative abundance of core OTUs in samples from each group. Normality and homogeneity of variance tests were first conducted on the data. One-way analysis of variance (One-way ANOVA) was applied for normally distributed data, while the Kruskal–Wallis non-parametric test was used for non-normally distributed data. A significance level of *p* < 0.05 was considered statistically significant. Box plots were employed to visualize the inter-group distribution of the total relative abundance of core OTUs. Box plots can intuitively reflect the median, upper and lower quartiles, dispersion, extreme values, and outliers of each group, clearly revealing the distribution differences and statistical characteristics of core microbiota abundance among different groups.

Microbial alpha diversity indices were calculated with Mothur software (Version 1.21.1, https://mothur.org/wiki/calculators/) (accessed on 8 May 2026)), and inter-group differences in the index values were further analyzed. The rarefaction analysisbased on Mothur v.1.21.1 [37] was conducted to reveal the diversity indices, including the Chao, ACE, and Shannon diversity indices. Beta diversity analysis was implemented based on the UniFrac algorithm [38]. Results of principal coordinate analysis (PCoA) were compared using the R-forge community ecology package, and non-metric multidimensional scaling (NMDS) was completed with the vegan package in R language. To identify microbial biomarkers with high-dimensional differences, LEfSe analysis was performed [39], and the effect size of each significantly enriched taxon was determined via linear discriminant analysis (LDA) [40]. Functional prediction of the microbial community was carried out using PICRUSt software (Version 1.1.09, http://picrust.github.io/picrust/) (accessed on 28 March 2026)). The OTU abundance table was mapped to Greengenes OTU IDs, and functional pathway annotation information at KEGG Level 1, Level 2 was obtained based on the KEGG database, http://www.genome.jp/kegg/ (accessed on 8 May 2026)), followed by the calculation of the relative abundance of each functional category. All statistical analyses and chart plotting were completed using OriginPro 2024 software based on the sequencing data.

## 3. Results

### 3.1. General Profile of 16S rRNA Sequencing Data

After raw sequencing of 5 sample groups from the intestinal tract of earwigs, a total of 1,151,202 high-quality reads with an average length of 422 bp were obtained. OTU clustering was performed on non-repetitive sequences (excluding single sequences) with 98.65% similarity, and species annotation results were statistically analyzed, yielding 34 phyla, 74 classes, 171 orders, 315 families, 739 genera, and 344 species (Table 1).

**Figure 2:**
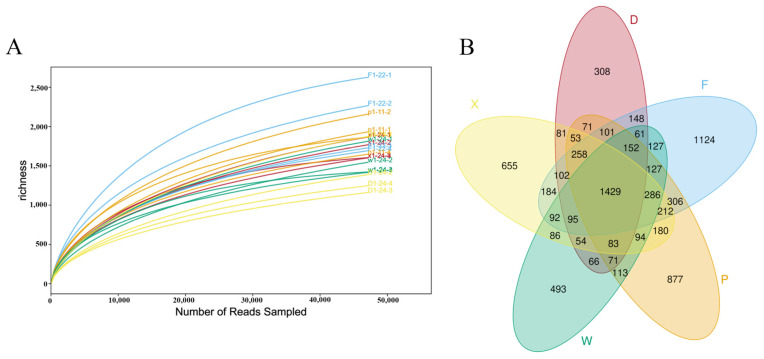
Rarefaction curves and Venn diagram of gut microbiota in earwigs. (**A**) Rarefaction curves of gut microbiota (**B**) Venn diagram of gut microbiota. Abbreviations in the figure represent different groups’ names: X, F, P, W, D. Four replicates were randomly selected from each group for sequencing, and the sample IDs were designated as follows: Group X: X-24-1, X-24-2, X-24-3, X-24-4; Group F: F-22-1, F-22-2, F-22-3, F-22-4; Group P: P-11-1, P-11-2, P-11-3, P-11-4; Group W: W-24-1, W-24-2, W-24-3, W-24-4; Group D: D-24-1, D-24-2, D-24-3, D-24-4.

The rarefaction curves indicated that the number of species tended to stabilize as the number of sequence reads increased, suggesting an appropriate sequencing depth (Figure 2A). There were significant differences in the number of OTUs in the gut microbiota among different earwig groups. Across all five sampling sites, a total of 1429 core OTUs were shared, accounting for 17.67% of the total OTUs (Figure 2B).

The specific OTUs quantity and unique OTUs distribution of each group were as follows: Group X: 3480 OTUs in total, including 655 unique OTUs (accounting for 8.10%); Group F: 4804 OTUs in total, with 1124 unique OTUs (accounting for 13.90%); Group P: 4413 OTUs in total, having 877 unique OTUs (accounting for 10.84%); Group W: 3429 OTUs in total, with 493 unique OTUs (accounting for 6.09%); Group D: 3597 OTUs in total, including 308 unique OTUs (accounting for 3.81%). The number of shared OTUs among the three cornfield groups (F, P, W) of the two earwig species was 565, while 726 OTUs were shared between the two mulberry orchard groups (D, X). Among conspecific individuals, 498 OTUs were shared among the three *T. komarovi* groups (F, P, X) from both habitats, and 582 OTUs were shared between the two *E. metallica* groups (D, W). Moreover, 1442 OTUs were shared between Groups F and P.

**Figure 3:**
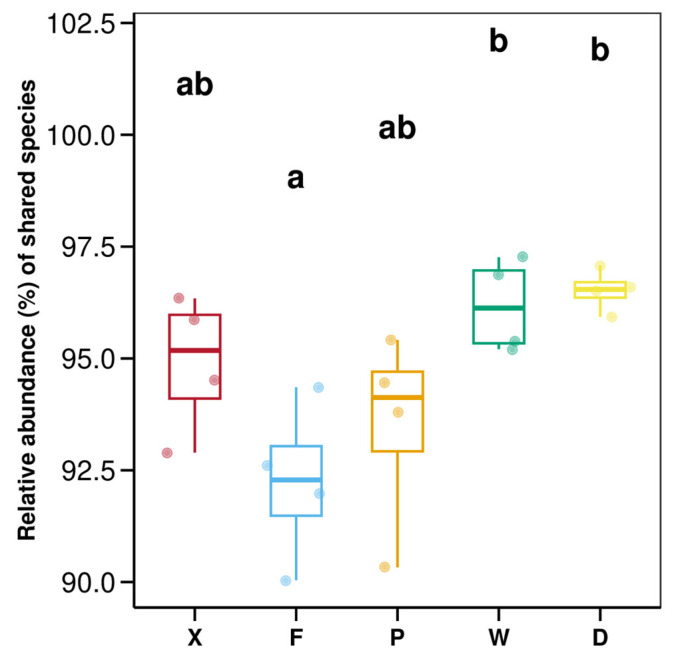
Shared species abundance diff_jitter. Abbreviations in the figure represent different groups’ names: X, F, P, W, D. Group X, *T. komarovi*, Mulberry; Group F, *T. komarovi*, cornfield; Group P, *T. komarovi*, cornfield; Group W, *E. metallica*, cornfield; Group D, *E. metallica*, Mulberry. Lowercase letters above the boxes indicate significant differences between different groups (one-way ANOVA, Tukey’s test, α = 0.05).

One-way ANOVA followed by Tukey’s post hoc multiple comparison was used to analyze inter-group differences in the relative abundance of shared species among the five groups (Figure 3). Results showed that the inter-group difference was statistically significant (*p* < 0.05 for the corresponding F-value). The box plot demonstrated that the relative abundance of shared species was significantly higher in group W and group D than in group F (*p* < 0.05). In contrast, group X and group P showed intermediate levels with no significant differences compared with other groups (*p* > 0.05).

### 3.2. Comparison of Gut Microbial Communities

**Figure 4:**
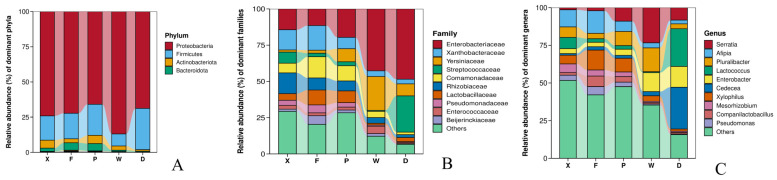
Composition of gut microbiota at the phylum (**A**), family (**B**), and genus (**C**) levels in different groups of earwigs. Abbreviations in the figure represent different groups’ names: X, F, P, W, D. Group X, *T. komarovi*, Mulberry; Group F, *T. komarovi*, cornfield; Group P, *T. komarovi*, cornfield; Group W, *E. metallica*, cornfield; Group D, *E. metallica*, Mulberry.

The community composition and relative abundance of gut microbiota in earwigs, the two species, *Timomenus komarovi* (Semenov, 1901) and *Eudohrnia metallica* (Dohrn, 1865) are shown in Figure 4. At the phylum level (Figure 4A), the top five taxa were Proteobacteria, Firmicutes, Actinobacteriota, Bacteroidota, and Myxococcota. The dominant and sub-dominant phyla were consistent across all five groups. Proteobacteria was the predominant phylum in Groups X, F, P, W, and D, with relative abundances of 74.10%, 72.27%, 65.86%, 86.90%, and 68.75%, respectively. Firmicutes was the sub-dominant phylum in all groups, with relative abundances of 17.25%, 18.12%, 22.04%, 8.55%, and 29.31%, respectively.

At the family level (Figure 4B), the top five taxa were Enterobacteriaceae, Xanthobacteraceae, Yersiniaceae, Streptococcaceae, and Comamonadaceae. Enterobacteriaceae was the dominant family in Groups X, P, W, and D, with relative abundances of 14.38%, 19.61%, 42.71%, and 48.49%, respectively. In contrast, Group F was dominated by Xanthobacteraceae (16.95%). Comamonadaceae was the sub-dominant family in both Groups F and P, accounting for 14.63% and 10.51%, respectively. The sub-dominant family in Group X was Rhizobiaceae (14.34%); in Group W, it was Yersiniaceae (23.37%); and in Group D, it was Streptococcaceae (25.00%).

At the genus level (Figure 4C), the top five genera were *Serratia*, *Afipia*, *Pluralibacter*, *Lactococcus*, and *Enterobacter*. Distinct differences in dominant genera were observed among groups. In Group X, *Afipia* (11.35%) was the most abundant genus, followed by *Lactococcus* (7.44%). Group P exhibited a relatively even distribution of genera, with *Pluralibacter* (9.38%) being the most abundant. Group F was mainly dominated by *Afipia* (14.97%) and *Xylophilus* (13.18%). Group W was predominated by *Serratia* (23.34%) and *Pluralibacter* (15.78%). In Group D, *Cedecea* (27.73%) and *Lactococcus* (24.98%) constituted the absolutely dominant genera.

### 3.3. Diversity of Gut Microbiota

#### 3.3.1. Analysis of Gut Microbial Alpha Diversity

**Figure 5:**
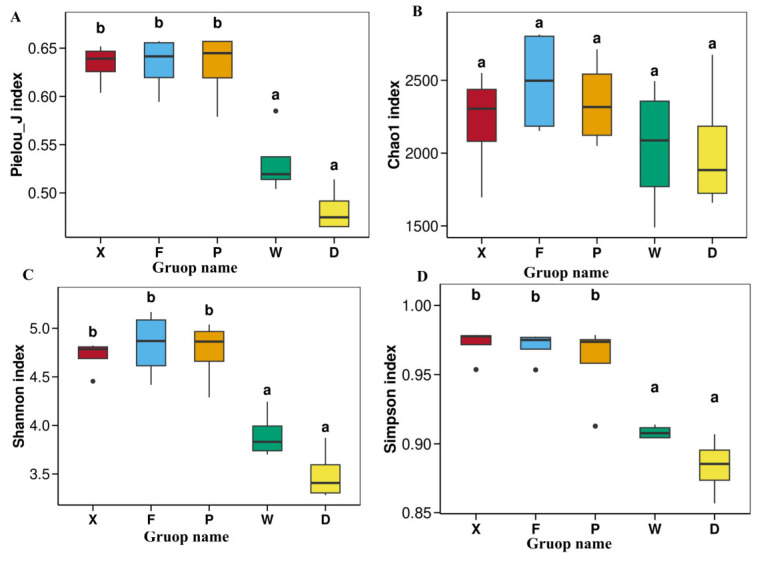
Box plots of gut microbial diversity of earwing fed on different host plants. (**A**) Pielou_J, (**B**) Chao1, (**C**) Shannon, (**D**) Simpson. Abbreviations in the figure represent different groups’ names: X, F, P, W, D. Group X, *T. komarovi*, Mulberry; Group F, *T. komarovi*, cornfield; Group P, *T. komarovi*, cornfield; Group W, *E. metallica*, cornfield; Group D, *E. metallica*, Mulberry. Lowercase letters above the boxes indicate significant differences between different gruops (one–way ANOVA, Tukey’s test, α = 0.05).

Alpha diversity indices reflect the species richness and diversity of gut microbial communities. An analysis was conducted on the alpha diversity indices of the gut microbiota in the two earwig species, *Timomenus komarovi* (Semenov, 1901) and *Eudohrnia metallica* (Dohrn, 1865), from five different habitats (Figure 5). as well as the inter-group *p*-values from significance tests (Table 2), with *p* < 0.05 defined as the threshold for significant differences.

The Pielou index (Figure 5A) was significantly higher in groups X, F, and P (marked with b). This indicates that the species distribution of gut microbiota in these three groups was the most even. Groups W and D (marked with a) showed significantly lower Pielou index values, suggesting a low evenness of microbial communities.

For species richness based on the Chao1 index (Figure 5B), Group F had the highest index value, indicating the highest number of gut microbial species. The Chao1 values gradually decreased in the order of X, P, W, and D. All groups were labeled with the same letter “a”, implying no statistically significant differences among the groups.

For comprehensive diversity based on the Shannon index (Figure 5C), groups X, F, and P (marked with b) exhibited significantly higher values. This reflects the highest comprehensive gut microbial diversity, with both high species richness and evenness. Groups W and D (marked with a) had significantly lower Shannon values, indicating obviously lower comprehensive diversity, accompanied by poor species richness and evenness.

For dominance and evenness reflected by the Simpson index (Figure 5D), groups X, F, and P (marked with b) showed significantly higher values, representing more evenly distributed species and weaker dominance of dominant taxa. Groups W and D had significantly lower Simpson values, indicating that a small number of dominant species occupied a higher proportion, and community evenness was poor.

Inter-group difference analysis showed that all pairwise *p*-values of the Chao1 richness index were much higher than 0.05 (ranging from 0.48 to 1.00), indicating no significant differences among any groups. This suggested that the total species richness of gut microbiota was basically consistent across the five groups (F, D, P, W, X), and environmental factors did not change the community species richness. Regarding the Shannon, Pielou, and Simpson indices, which reflect diversity, evenness, and dominance, respectively, the three *T. komarovi* groups (X, P, F) were significantly different from the two *E. metallica* groups (W, D) (*p* < 0.05). Meanwhile, no significant differences were observed within each species group (*p* > 0.05).

#### 3.3.2. Analysis of Gut Microbial Beta Diversity

**Figure 6:**
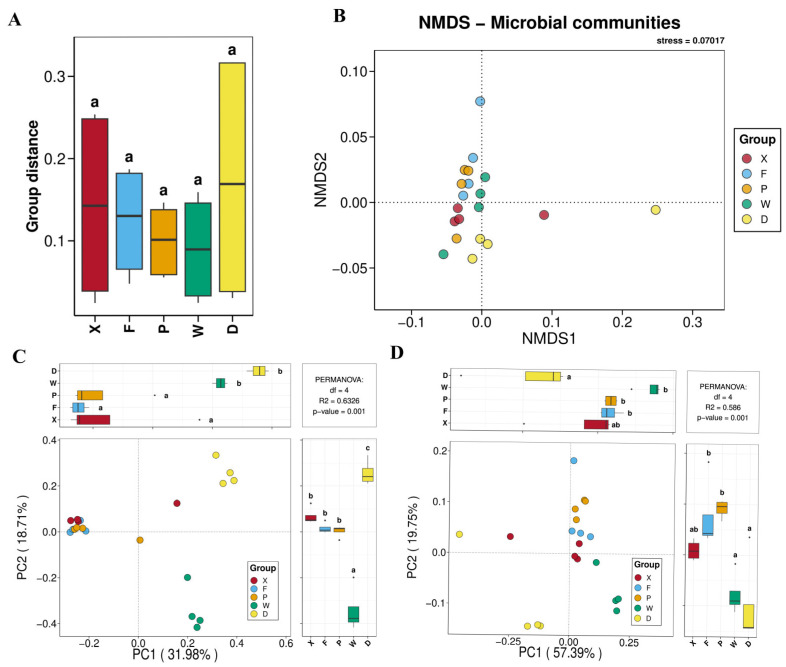
Beta diversity analysis of gut microbiota in earwigs. (**A**) Inter-sample distance analysis (**B**) NMDS analysis, ordination of microbial communities (stress = 0.07017); (**C**) PCoA analysis based on the Bray–Curtis algorithm, with PERMANOVA results (df = 4, R^2^ = 0.6326, *p* = 0.001); (**D**) PCoA analysis based on the weighted UniFrac algorithm, with PERMANOVA results (df = 4, R^2^ = 0.588, *p* = 0.001). Different lowercase letters (a, b, c, ab) above or beside the bars indicate significant differences (*p* < 0.05) based on post-hoc multiple comparisons. Bars sharing the same letter are not significantly different, while different letters denote statistically significant differences between groups. Abbreviations in the figure represent different groups’ names: X, F, P, W, D. Group X, *T. komarovi*, Mulberry; Group F, *T. komarovi*, cornfield; Group P, *T. komarovi*, cornfield; Group W, *E. metallica*, cornfield; Group D, *E. metallica*, Mulberry.

The results of the analysis of differences in gut microbial community structure among earwig groups are presented in Figure 6. Inter-group differences were examined using Tukey’s HSD test. As shown in the boxplot (Figure 6A), the microbial community structure in the cornfield groups (F, P, W) demonstrated greater stability, consistency, and repeatability than that in the mulberry orchard groups (D, X). No significant differences were observed between the groups. NMDS analysis (Figure 6B) revealed that the community structure of group X was the most stable; groups F and P showed high similarity in community structure; group W was clearly distinct from groups X, F, and P; and group D exhibited the most significant separation from the other four groups. PCoA analysis (Figure 6C,D) was conducted based on the Bray–Curtis and weighted UniFrac algorithms, the patterns were highly consistent with the NMDS results.

The above results indicated that with regard to the gut microbial community structure, the three groups of *T. komarovi* (X, F, and P groups) showed small intra-group differences, but exhibited distinct inter-group differences when compared with *E. metallica* (W and D groups). Considerable differences were also observed between the W and D groups. Large community differences existed between mulberry orchard habitats (X and D groups) and cornfield habitats (F, P, and W groups). For the same earwig species within the same habitat across different geographical regions, the F and P groups displayed minor differences.

It is evident that host species specificity plays a decisive role in shaping the gut microbial community structure of earwigs and dominates community differentiation among different host species. Meanwhile, habitat type acts as a key environmental filtering factor, exerting a significant differentiating effect on the microbial community composition within the same earwig species. In addition, conspecific earwigs from different geographical regions exhibited a high degree of community similarity, implying that the gut microbiota possesses strong stability and conservatism within host species, and geographical location has only a weak influence on gut microbial communities.

#### 3.3.3. Heatmap of the Top 30 Most Abundant Genera

**Figure 7:**
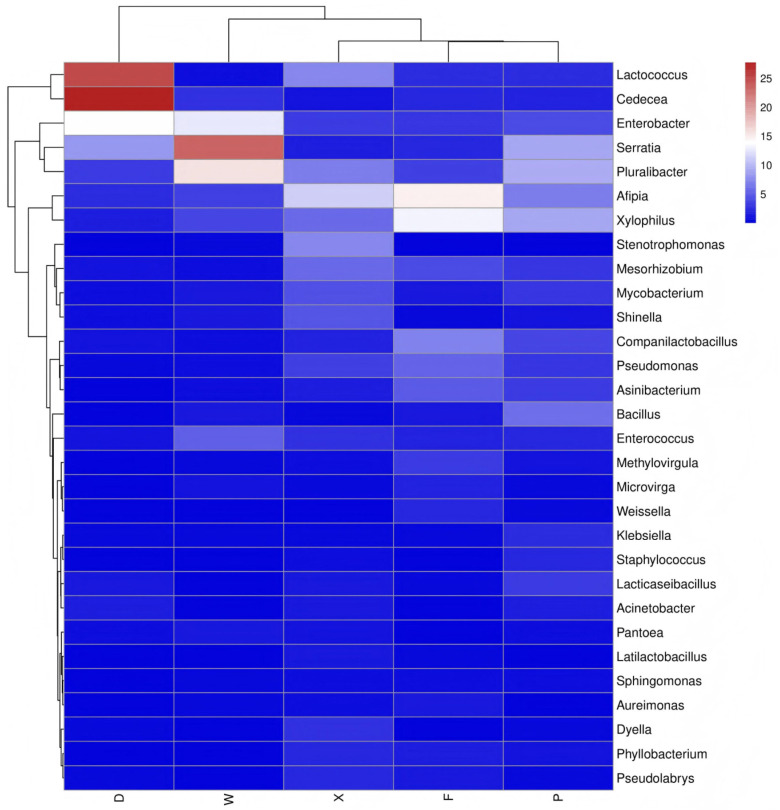
Heatmap of the top 30 most abundant genera in the different groups of earwigs from different species and habitats. The columns represent the samples and rows represent the bacteria at the genus level. The color scale represents the normalized values of relative abundances by log10. Abbreviations in the figure represent different groups’ names: X, F, P, W, D. Group X, *T. komarovi*, Mulberry; Group F, *T. komarovi*, cornfield; Group P, *T. komarovi*, cornfield; Group W, *E. metallica*, cornfield; Group D, *E. metallica*, Mulberry.

As shown in the heatmap based on the top 30 most abundant genera (Figure 7), the gut microbiota of the five earwig groups were clustered into two major clades according to host species: *E. metallica* (groups D and W) and *T. komarovi* (groups X, F, and P). Group D was dominated by *Lactococcus* and *Cedecea* as the absolutely dominant genera, while Group W was characterized by *Serratia* as the core genus. *T. komarovi* groups (X, P, F) harbored *Afipia*, *Pluralibacter*, and *Xylophilus* as indicator genera. Groups F and P (conspecific, different geographical regions and in the same habitat) were clustered most closely and exhibited the highest similarity in microbial community.

#### 3.3.4. LEfSe Analysis of Gut Microbial Taxa

**Figure 8:**
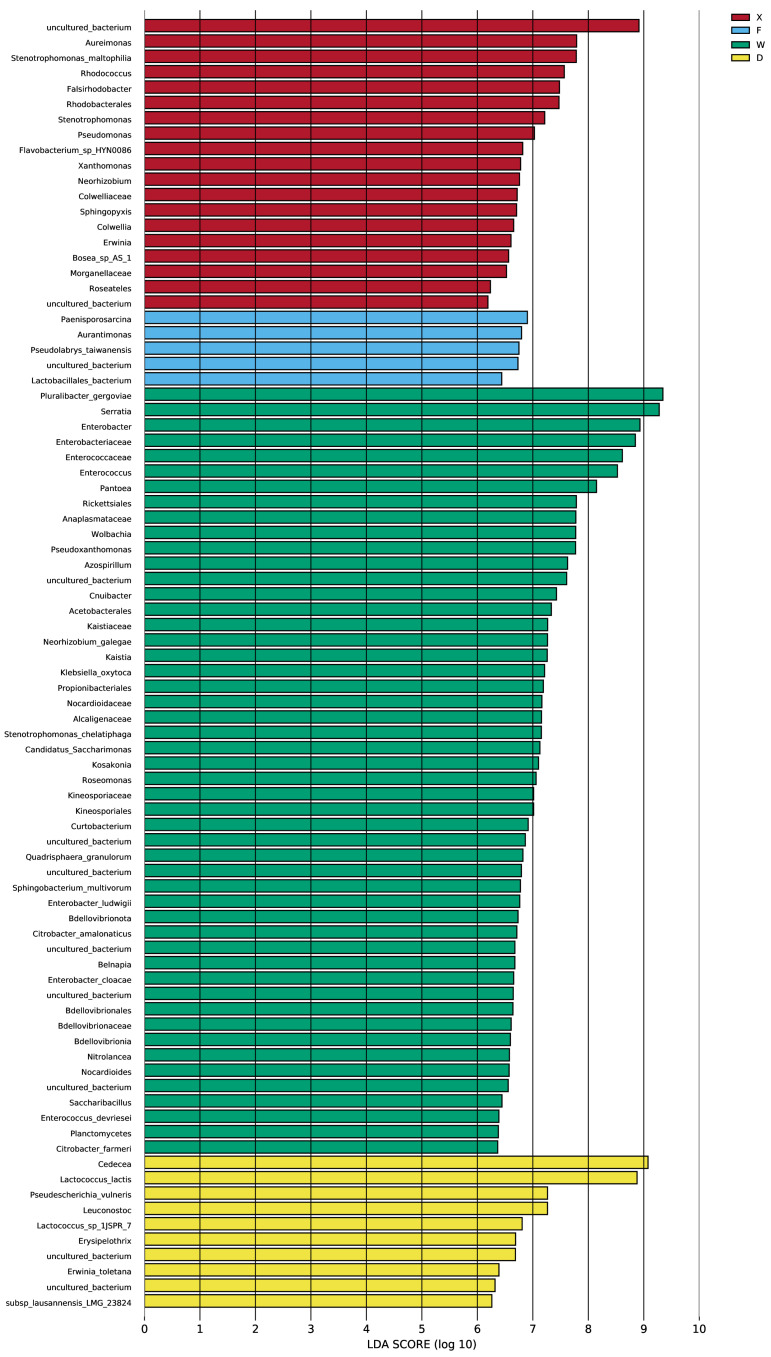
LEfSe analysis of gut microbial taxa in earwigs of different species from different habitats. Histogram of LDA value distribution. Abbreviations in the figure represent different groups’ names: X, F, P, W, D. Group X, *T. komarovi*, Mulberry; Group F, *T. komarovi*, cornfield; Group P, *T. komarovi*, cornfield; Group W, *E. metallica*, cornfield; Group D, *E. metallica*, Mulberry.

**Figure 9:**
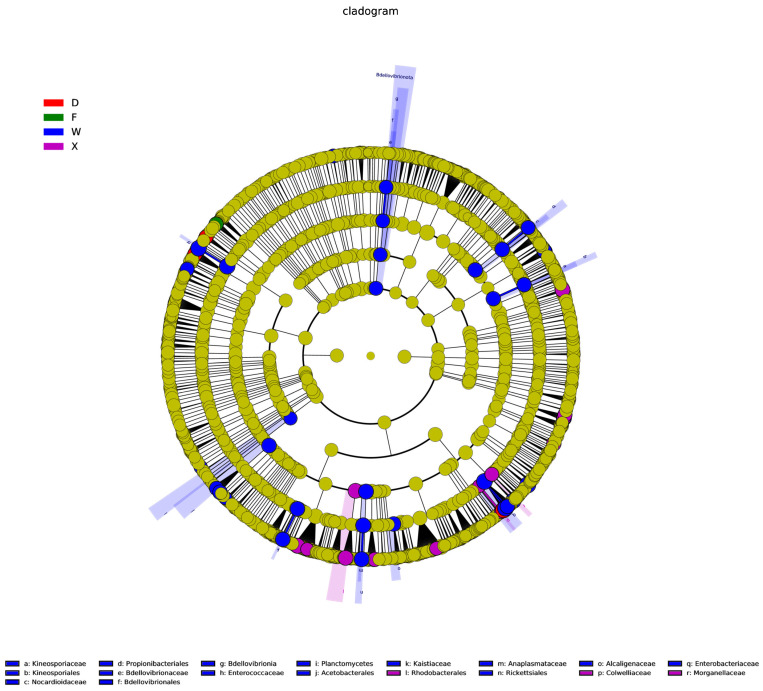
LEfSe analysis of gut microbial taxa in earwigs of different species from different habitats. Evolutionary cladogram. The radiating circles from the inside to the outside in the figure represent the taxonomic levels from Phylum to Genus (or Species). Each small circle at different taxonomic levels stands for a taxon at that level, and the diameter of the small circles is directly proportional to the relative abundance. Coloring principle: Species with no significant differences are uniformly colored yellow; differential species biomarkers are colored according to their corresponding groups. Red nodes indicate microbial taxa that play an important role in the red group, and green nodes indicate microbial taxa that play an important role in the green group. The species names marked by English letters in the figure are shown in the legend on the right. Abbreviations in the figure represent different groups’ names: X, F, P, W, D. Group X, *T. komarovi*, Mulberry; Group F, *T. komarovi*, cornfield; Group P, *T. komarovi*, cornfield; Group W, *E. metallica*, cornfield; Group D, *E. metallica*, Mulberry.

In this study, the LEfSe method was employed to analyze the bacterial community structure at multiple taxonomic levels (from phylum to genus) with an LDA threshold of 4 (Figure 8). An evolutionary cladogram was further constructed to characterize the divergence of bacterial communities under different sampling conditions (Figure 9). The results revealed significant differences in gut microbial community composition among habitats and earwig species. As shown in Figure 8, Group W exhibited the most diverse set of differential taxa, with 50 biomarkers distributed across 7 phyla and 16 orders, including functionally diverse groups such as Actinobacteriota and Bacteroidota. Group X contained 19 differential biomarkers belonging to 4 phyla and 10 orders, dominated by α-Proteobacteria and γ-Proteobacteria of the phylum Proteobacteria. Group D had 10 differential biomarkers from 2 phyla and 3 orders, with Enterobacterales as the primary component. Group F possessed 5 differential biomarkers from 2 phyla and 4 orders, mainly represented by Bacillales and Rhizobiales. No significantly different microorganisms were detected in Group P.

### 3.4. PICRUSt2 Functional Prediction of Earwig Gut Microbiota

**Figure 10:**
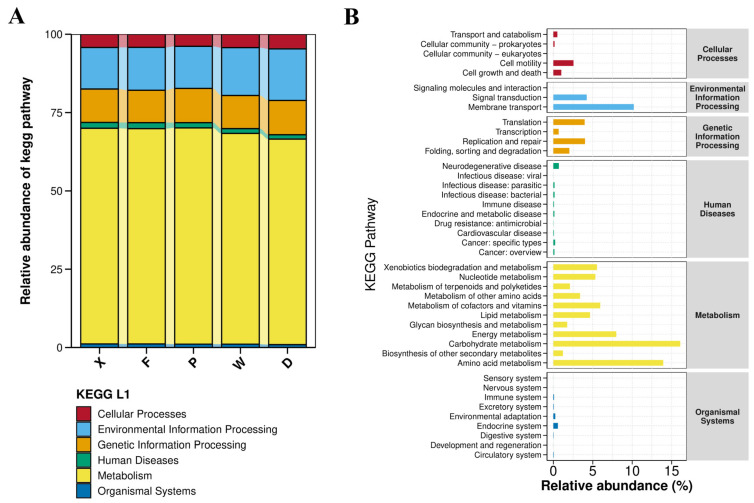
KEGG pathway abundance statistics of gut microbiota in earwigs. (**A**) Stacked bar chart of relative abundance for PICRUSt functional annotation at KEGG Level 1; (**B**) Bar chart of KEGG pathway abundance. Abbreviations in the figure represent different groups’ names: X, F, P, W, D. Group X, *T. komarovi*, Mulberry; Group F, *T. komarovi*, cornfield; Group P, *T. komarovi*, cornfield; Group W, *E. metallica*, cornfield; Group D, *E. metallica*, Mulberry.

Gene functional annotation was used to preliminarily characterize the functional profiles of gut microbial communities in earwigs. Based on the KEGG database annotation, the stacked bar chart of KEGG Level 1 functions (Figure 10A) showed that the functional genes of earwig gut microbiota were categorized into six primary pathways. Among them, metabolism was the most highly enriched (67.92%), followed by environmental information processing (14.43%), genetic information processing (10.69%), cellular processes (4.23%), human diseases (1.67%), and organismal systems (1.05%, the lowest). The bar chart of KEGG Level 2 functional abundance (Figure 10B) revealed that the functional genes were classified into 42 secondary pathways. The top 10 most abundant pathways were: carbohydrate metabolism (16.09%), amino acid metabolism (13.93%), membrane transport (10.20%), energy metabolism (7.98%), metabolism of cofactors and vitamins (5.95%), xenobiotics biodegradation and metabolism (5.53%), nucleotide metabolism (5.33%), lipid metabolism (4.65%), signal transduction (4.23%), and replication and repair (4.01%). Among these, seven pathways were related to metabolism, two to environmental information processing, and one to genetic information processing.

**Figure 11:**
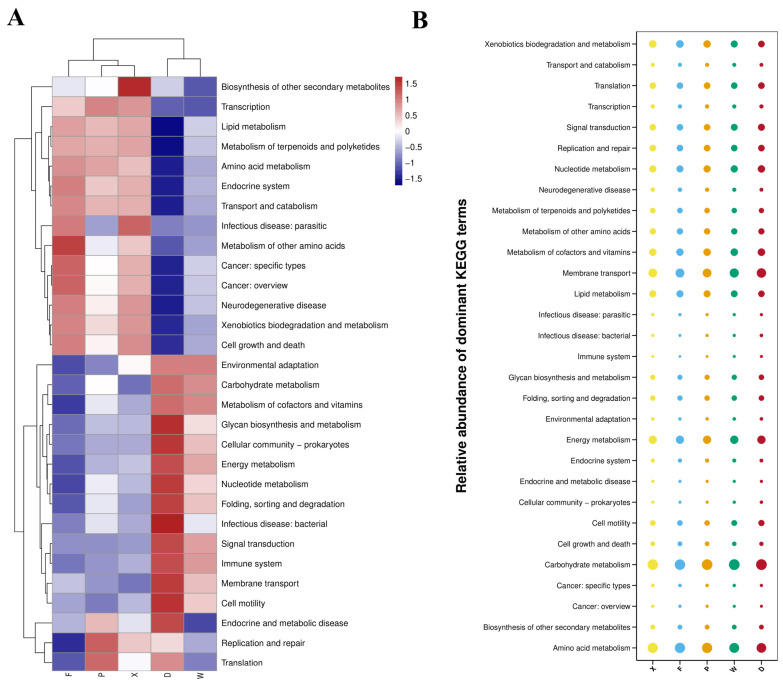
(**A**) Cluster heatmap of PICRUSt functional annotation at KEGG Level 2; (**B**) Bubble plot of relative abundance for PICRUSt functional annotation at KEGG Level 2. Abbreviations in the figure represent different groups’ names: X, F, P, W, D. Group X, *T. komarovi*, Mulberry; Group F, *T. komarovi*, cornfield; Group P, *T. komarovi*, cornfield; Group W, *E. metallica*, cornfield; Group D, *E. metallica*, Mulberry.

The KEGG Level 2 functional clustering heatmap (Figure 11A) showed that these two clades, together with the *T. komarovi* Group F, formed a single large cluster, indicating that the host genetic background plays a decisive role in shaping the functional profiles of the gut microbiota. In the cornfield habitat, Groups F and P were more closely clustered, whereas Group W was relatively distant. This reflects the functional conservatism dominated by host species, with the habitat exerting only a fine-tuning effect. In the mulberry orchard habitat, Groups X and D formed independent clusters, revealing significant differences in functional adaptation strategies between the two species under the highly stressful mulberry environment. For the *T. komarovi* groups (X, P, F), the relative abundance of functional genes at the secondary pathway level exhibited habitat-specific differentiation. Group F showed a higher abundance in pathways related to other amino acid metabolism and cancer-related functions. Group X was dominant in the biosynthesis of other secondary metabolites and pathways associated with infectious parasitic diseases. Group P displayed a higher abundance in pathways related to translation, replication and repair, and transcription. In the *E. metallica* groups (D, W), differences in the abundance of secondary pathways were even more pronounced. Group D exhibited a higher abundance in multiple pathways, including glycan biosynthesis and metabolism, prokaryotic communities, and energy metabolism. In contrast, Group W was enriched in pathways such as environmental adaptation, carbohydrate metabolism, and metabolism of terpenoids and polyketides. These results suggest that the functions within the *T. komarovi* groups undergo directional specialization across habitats, whereas the functions of *E. metallica* show a more extreme environmental response and stronger plasticity.

The KEGG Level 2 functional bubble plot (Figure 11B) revealed a distinct pattern of core-dominated, gradually distributed functional abundance in the gut microbiota of earwigs. Carbohydrate metabolism (15.55–16.66%), amino acid metabolism (12.40–14.79%), and membrane transport (9.38–11.54%) represented the highly abundant core functions shared across all groups. Medium-to-low abundance functions, such as energy metabolism, metabolism of cofactors and vitamins, and xenobiotics biodegradation and metabolism, decreased in an orderly gradient from approximately 8% to 0.1%, reflecting the integrity and systematic nature of the functional system of earwig gut microbiota. Inter-group comparative analysis demonstrated significant differences in functional bubble patterns between the *T. komarovi* groups (F, P, X) and the *E. metallica* groups (W, D). The *E. metallica* groups exhibited significantly higher abundance of carbohydrate metabolism and membrane transport functions, whereas the *T. komarovi* groups were more prominent in amino acid metabolism, xenobiotic biodegradation and metabolism, and lipid metabolism. Within the same species, Groups F and P from different habitats only showed minor variations in core functional abundance, and the functional profiles within the *T. komarovi* groups were highly consistent, highlighting stronger functional conservatism.

## 4. Discussion

### 4.1. Discussion on Overview of 16S rRNA Sequencing Data

This study revealed that the gut microbiota of these two earwig species is centered on 1429 core shared OTUs, exhibiting a hierarchical assembly pattern of a “core-specialized” structure. There is obvious differentiation in the gut microbial composition between these two earwig species, and they have formed their own unique microbial taxa. One-way ANOVA combined with Tukey’s multiple comparison analysis showed significant inter-group differences in the relative abundance of shared species. It is proposed that the habitat microenvironment and food resources can substantially influence the enrichment level of core shared microbial communities.

### 4.2. Analysis and Discussion of Differences in Bacterial Community Composition

Earwig gut microbiota exhibit high conservation at the phylum level, with a stable composition of dominant and sub-dominant bacterial phyla. However, microbial community differentiation becomes increasingly significant as the taxonomic resolution is refined to the family and genus levels. The stable existence of dominant taxa such as Proteobacteria, Firmicutes, Enterobacteriaceae, and *Serratia* provides microbial functional support for nutrient digestion, environmental adaptation, and stress resistance of earwigs. The relatively fixed composition of dominant phyla and core genera in earwig gut microbiota is highly consistent with the dominant microbial groups of most predatory natural-enemy insects [41]. This not only reflects the evolutionary convergence of gut microbial community composition among insects [10,42], but also indicates that the core functions of earwig gut microbiota possess strong stability and availability as natural-enemy insects [9,40,43]. The specifically enriched bacterial families and genera in each group are important microbial indicators for adapting to microecological conditions of different habitats. They lay a microecological foundation for earwigs to adapt to heterogeneous agricultural habitats and endow them with development potential as natural-enemy insects [9,10,15].

### 4.3. Discussion on the Analysis of Gut Microbial Diversity Structural and Functional Adaptation of Earwig Gut Microbiota to Habitats

Diversity and genus-level analyses indicated that the microbiota of earwigs exhibited stable species richness but pronounced divergence in community structure. This suggests that the evaluation of their ecological functions should focus on the relative abundance of dominant taxa.

Host species and habitat type were identified as key factors shaping the earwig gut microbiota. Specifically, the gut microorganisms of *T. komarovi* and *E. metallica* showed obvious host specificity in terms of diversity, evenness, and functional composition. Among the two species, *T. komarovi* had significantly higher gut microbial diversity, endemism, and community stability than *E. metallica*. In terms of habitat effects, cornfield habitats tended to promote higher microbial diversity, whereas the mulberry orchard environment led to greater microbiota homogenization. This is likely due to the stronger environmental stress in mulberry orchards, which filtered out specific taxa and reduced inter-individual microbial differences. The strong stability of gut microbiota within the same earwig species provides a crucial microecological foundation for their large-scale development and application across different regions. This phenomenon reflects the evolutionary convergence in the community composition of insect gut microbiota [11,36], and further indicates that the core functions of earwig gut microbiota possess high stability, endowing earwigs with exploitable potential as biocontrol agents [10,12,38].

Two complementary analytical methods (LEfSe and heatmap) were used to verify the differentiation characteristics of gut microbiota. LEfSe analysis accurately identified microbial biomarkers for each group, and the quantitative gradient of these biomarkers reflected the degree of specialization and stability of earwigs’ adaptation to their habitats. Notably, Group P exhibited high homeostatic characteristics, which can serve as an ideal reference for the large-scale artificial rearing of earwigs. Additionally, various biomarkers were highly adapted to their corresponding habitat characteristics, making them potential indicators of habitat adaptability and targets for microecological regulation.

Heatmap analysis of the top 30 genera in relative abundance further confirmed that earwig gut microbiota clustered obviously according to host species. Samples from the same host species and the same habitat showed the highest similarity in microbial composition, while different hosts and groups had their own signature dominant genera, indicating obvious differentiation in microbial community structure. This result further supports the conclusion that host species is a core driver of microbial community differentiation.

KEGG functional pathway analysis verified the adaptive pattern of “conserved core functions coupled with habitat-specialized differentiation” in these two earwig gut microbiota. Metabolic pathways constituted the core functions with a stable basic functional framework. Host species drove the core differentiation of microbial functions, whereas habitat mediated fine-tuning of functional profiles. The cornfield groups displayed higher functional abundance, providing a theoretical basis for optimizing artificial diet formulations.

### 4.4. PICRUSt2-Based Predicted Functional Characteristics of Earwig Gut Microorganisms and Implications for Natural Enemy Utilization

The functional structure of earwig gut microorganisms is centered on basal metabolism and maintains a high degree of conservation. Host species dominate the differentiation of functional composition, while habitat only mediates the fine-tuning and specialization of partial specific pathways. The two earwig species form a gut microbial functional regulation strategy adapted to different agricultural habitats through a pattern of constant core functions and habitat-specific adaptation of accessory functions. The gut microbiota of earwigs represents an ordered symbiotic system jointly shaped by host genetics and environmental selection [10,12]. Habitat differences between mulberry orchards and maize fields impose directional screening: the gut microbiota of *E. metallica* undergoes structural alteration, while *T. komarovi* only slightly adjusts microbial abundance, showing stronger adaptability and flexibility.

Based on the above findings, a differentiated development strategy can be implemented for earwigs as natural enemy insects. With strong habitat adaptability, *T. komarovi* can be preferentially developed as a broad-spectrum natural enemy, while *E. metallica* is suitable for targeted development focusing on its specific adaptive habitats. Meanwhile, gut microbial biomarkers can be applied to optimize artificial rearing and field application strategies. The conservation of core microbial functions can be utilized to establish a standardized system for large-scale rearing. Meanwhile, localized adaptation can be realized by considering regional microenvironmental characteristics. Protecting and enriching beneficial gut microbiota can improve the field colonization ability and pest control efficiency of earwigs, providing important microecological theoretical support and practical reference for the conservation and efficient utilization of natural enemy insect resources.

## 5. Conclusions

### 5.1. Clarification of the Composition and Structural Characteristics of Earwig Gut Microbes

This study indicated that the dominant phyla of these two earwig gut microorganisms are Proteobacteria and Firmicutes. The dominant families include Enterobacteriaceae, Xanthobacteraceae, Yersiniaceae, Streptococcaceae, and Comamonadaceae. The dominant genera are *Serratia*, *Afipia*, *Pluralibacter*, *Lactococcus*, and *Enterobacter*, with differences in dominant genera among different groups.

### 5.2. The Regulatory Factors and Adaptive Characteristics of Gut Microbial Diversity in Two Earwig Species Were Clarified

The gut microorganisms of *T. komarovi* and *E. metallica* indicate that the species richness of the gut microbial community is conservative, while community diversity, evenness, and dominance are mainly regulated by the host species. The overall gut microbial community exhibits strong stability and conservatism. Habitat type has a significant differentiating effect on the microbial community composition, whereas the influence of the geographical region is negligible. The impact of habitat type on gut microorganisms is reflected in the fact that the stability and sample repeatability of the microbial community in the cornfield habitat are superior to those in the mulberry orchard habitat.

### 5.3. Elucidation of the Functional Characteristics of Earwig Gut Microbes

The functional structure of *T. komarovi* and *E. metallica* gut microorganisms is centered on basal metabolism and maintains a high degree of conservation. Host species dominate the differentiation of functional composition, while habitat only mediates the fine-tuning and specialization of partial specific pathways. The functional genes of gut microorganisms are classified into six major categories, among which metabolic genes occupy the highest abundance (67.92%). The core metabolic functions are mainly concentrated in carbohydrate metabolism, amino acid metabolism, and energy metabolism.

### 5.4. Revealing the Potential of the Two Earwig Species for Development as Natural Enemies

Significant differences exist in the gut microbial adaptation strategies of *T. komarovi* and *E. metallica*, which determine their distinct development potential and application scenarios as natural enemy insects. *T. komarovi* has high community stability and wide habitat adaptability, making it suitable for development as a broad-spectrum natural enemy. *E. metallica* shows a high degree of community specialization and host specificity and is applicable for targeted development. In addition, the dominant gut microbiota and specific biomarkers of earwigs can be used as microecological regulation targets for the development of natural enemies.

## Figures and Tables

**Table 1:** Basic Information of Third-Generation High-Throughput Sequencing of the 16S rRNA Gene for Enteric Microbiota in Lushuius.

Taxonomy	Phylum	Class	Order	Family	Genus	Species
Total	34	74	171	315	739	344
X-24-1	16	32	86	147	300	134
X-24-2	15	31	80	138	272	126
X-24-3	21	38	89	152	291	116
X-24-4	15	31	66	114	230	117
F-22-1	22	49	112	176	362	151
F-22-2	20	43	99	163	327	153
F-23-3	17	32	75	134	267	124
F-23-4	18	37	81	135	279	110
P-11-1	18	39	93	156	303	139
P-11-2	20	39	94	158	308	149
P-11-33	21	45	100	169	334	144
P-11-4	19	32	69	115	235	123
W-24-1	18	34	86	139	286	127
W-24-2	15	26	70	122	247	120
W-24-3	19	38	85	143	269	97
W-24-4	13	26	62	106	218	117
D-24-1	16	33	83	138	265	128
D-24-2	15	26	69	107	193	85
D-24-3	13	21	52	88	167	69
D-24-4	15	27	59	103	189	89

Total: Total collected earwig samples. Abbreviations in Table 1 represent different gruops name: X, F, P, W, D: Group X, *T. komarovi*, Mulberry; Group F, *T. komarovi*; Group P, *T. komarovi*; Group W, *E. metallica*; Group D, *E. metallica*, Mulberry Orchards, the same applies below.

**Table 2:** Comparison of alpha index distributions of gut microbiota among different group of earwing (*p*-values).

*p*-Value	Groups F-D	Groups P-D	Groups W-D	Groups X-D	Groups P-F	Groups W-F	Groups X-F	Groups W-P	Groups X-P	Groups X-W
Chao1	0.4833	0.7745	1.0000	0.9589	0.9855	0.5125	0.8566	0.8010	0.9879	0.9689
Shannon	0.0000	0.0001	0.2839	0.0002	0.9968	0.0023	0.9731	0.0045	0.9989	0.0075
Simpson	0.0001	0.0003	0.3691	0.0001	0.9260	0.0023	0.9999	0.0105	0.8810	0.0017
Pielou_J	0.0000	0.0000	0.1820	0.0000	1.0000	0.0019	1.0000	0.0023	1.0000	0.0020

## Data Availability

All data presented in this study are included in this article. For further inquiries, please contact the first author.

## References

[1] Lippey M.K., Rosenheim J.A., Paredes D., Karp D.S., Emery S.E., Kramer R.C., Sharp R., Meineke E.K. (2025). One landscape does not fit all: Diverse arthropod responses to land use. Ecol. Appl..

[2] You L., Qiu X., Xiao T., Wang J., Chen Z. (1997). Study on *Allodahlia scabriuscula* in Reed Fields of Dongting Lake Region. Acta Entomol. Sin..

[3] Bortolotto O., Santos J., Ochonski A. (2024). First report of earwig *Doru luteipes* (Dermaptera: Forficulidae) preying on corn leafhopper *Dalbulus maidis* (Hemiptera: Cicadellidae) nymphs. Braz. J. Biol..

[4] Moreira L.B., Lima L.L.R., de Sá Farias E., Carvalho G.A. (2023). Response of *Doru luteipes* (Dermaptera: Forficulidae) to insecticides used in maize crop as a function of its life stage and exposure route. Environ. Sci. Pollut. Res..

[5] Liu H., Ning G., Li Z., Zhang L., Chen B. (2014). Predation of Adult *Timomenus komarowi* on Larvae and Pupae of *Ostrinia furnacalis*. Southwest China J. Agric. Sci..

[6] Tian C., Zhang J., Li G., Huang J., Yin X., Feng H. (2022). Biological Characteristics of *Labidura riparia* and Its Predation Capacity on *Spodoptera frugiperda*. J. Plant Prot..

[7] Tian C., Cao H., Zhang J., Liu X., Cai T., Li G., Huang J., Feng H. (2021). Predatory Behavior and Functional Response of Earwigs on *Spodoptera frugiperda*. Chin. J. Biol. Control.

[8] Tian C., Zhang J., Xu C., Li G., Huang J., Liu Y., Wang G., Feng H., Yin X., Feng H. (2023). Predation Capacity of *Labidura riparia* on *Helicoverpa armigera*. Plant Prot..

[9] Han S., Zhou Y., Wang D., Qin Q., Song P., He Y. (2023). Effect of Different Host Plants on the Diversity of Gut Bacterial Communities of *Spodoptera frugiperda* (J. E. Smith, 1797). Insects.

[10] Wang S., Qu S. (2017). Insect Symbiotic Microbes and Their Application Prospects in Pest and Disease Control. Bull. Chin. Acad. Sci..

[11] Douglas A.E. (2015). Multiorganismal insects: Diversity and function of resident microorganisms. Annu. Rev. Entomol..

[12] Jing T.Z., Qi F.H., Wang Z.Y. (2020). Most Dominant Roles of Insect Gut Bacteria: Digestion, Detoxification, or Essential Nutrient Provision?. Microbiome.

[13] Sun J., Meng Y., Chen Z., Zhao T., Yang C., Chen S., Wang J., Tian L., Song F., Duan Y. (2026). Gut microbiome convergence and functional adaptation underlie the evolution of predation in stink bugs (Heteroptera: Pentatomidae). Microbiome.

[14] Savio C., Mugo-Kamiri L., Upfold J.K. (2022). Bugs in bugs: The role of probiotics and prebiotics in maintenance of health in mass-reared insects. Insects.

[15] Yan X., Li J., Sun Z., Yan C. (2025). An Insight into Biology, Function and Pest Management Guidance of Gut Microbiota in *Spodoptera frugiperda*. Insects.

[16] Tiede J., Scherber C., Mutschler J., McMahon K.D., Gratton C. (2017). Gut microbiomes of mobile predators vary with landscape context and species identity. Ecol. Evol..

[17] Mikaelyan A., Dietrich C., Köhler T., Poulsen M., Sillam-Dussès D., Brune A. (2015). Diet is the primary determinant of bacterial community structure in the guts of higher termites. Mol. Ecol..

[18] Li H., Qiu M.Y., Zhang Y.X., Huang S.B., Liu H.Y., Xiao Y.S., Liu T., Jin F.L., Xu X.X. (2025). Environmental Factors Drive Significant Differences in Gut Microbiota Structure and Diversity of Female *Rhynocoris fuscipes*. Chin. J. Biol. Control..

[19] Sanz Y., Cryan J.F., Deschasaux T.M., Elinav E., Lambrecht R., Veiga P. (2025). The gut microbiome connects nutrition and human health. Nat. Rev. Gastroenterol. Hepatol..

[20] Segev T., Barak D., Zahavi L., Godneva A., Rein M., Krongauz D., Rossman H., Weinberger A., Segal E. (2025). Diet shapes the gut microbiome: Cross-sectional and longitudinal insights from the Human Phenotype Project. medRxiv.

[21] Zhang P. (2022). Influence of foods and nutrition on the gut microbiome and implications for intestinal health. Int. J. Mol. Sci..

[22] Su J., He K., Lian C., Zhang X., Yu K. (2022). Gut Microbiota Diversity of Wild Small Mammals in Yunnan Based on 16S rRNA Gene Amplicon Sequencing. Acta Sci. Nat. Univ. Pekin..

[23] Zhang M.Y. (2023). Mechanism of Gut Microbes in Dietary and Seasonal Adaptation of Non-Human Primates. Master’s Thesis.

[24] Ma L., Wang D., Ren Q., Sun J., Zhang L., Cheng Y., Jiang X. (2024). Gut Microbiota Affects Host Fitness of Fall Armyworm Feeding on Different Food Types. Insects.

[25] Li D., Li J., Hu Z., Liu T., Zhang S. (2022). Fall Armyworm Gut Bacterial Diversity Associated with Different Developmental Stages, Environmental Habitats, and Diets. Insects.

[26] Dong W., Hafeez M., Zhao S., Zhang J.-M., Imran M., Ullah F., Li X.-W., Lu Y.-B. (2025). Influence of Host’s Plant Diet on Gut Microbial Communities and Metabolic Potential in *Spodoptera frugiperda*. Insects.

[27] Jelena R., Jelena T., Marija R., Tanja L., Tatjana S., Bojan B., Biljana B.N., Pavković-Lučić S. (2025). Different Long-Term Nutritional Regimens of *Drosophila melanogaster* Shape Its Microbiota and Associated Metabolic Activity in a Sex-Specific Manner. Insects.

[28] Meng L., Xia C., Jin Z., Zhang H. (2022). Investigation of Gut Bacterial Communities of Asian Citrus Psyllid (*Diaphorina citri*) Reared on Different Host Plants. Insects.

[29] Zhang X., Yang H., Yan Z., Wang Y., Wang Q., Huo S., Chen Z., Cheng J., Yang K. (2025). Host-Dependent Variation in *Tetranychus urticae* Fitness and Microbiota Composition Across Strawberry Cultivars. Insects.

[30] Gong Q., Cao L., Sun L., Chen J.C., Gong Y.J., Pu D.Q., Huang Q., Hoffmann A.A., Wei S.J. (2020). Similar Gut Bacterial Microbiota in Two Fruit-Feeding Moth Pests Collected from Different Host Species and Locations. Insects.

[31] Zhu Y.F., Han R., Zhang T., Yang J.W., Teng Z.W., Fan Y.J., Sun P.D., Lu Y.Y., Ren Y.L., Wan F.H. (2024). The Food Source and Gut Bacteria Show Effects on the Invasion of Alien Pests: A Case of *Bactrocera dorsalis* (Hendel) (Diptera: Tephritidae). Insects.

[32] Chen Q., Yi X., Wang X., Zheng X., Lu W. (2023). A Limiting Factor of Sex Attractants of *Bactrocera dorsalis* (Diptera: Tephritidae), Verified under Laboratory Conditions. Insects.

[33] Chen Y., Ma W. (2004). Fauna Sinica, Insecta. Vol. 35. Dermaptera.

[34] Li J., Sun Z., Tian X., Wang H., Zhou F., Shi C., Li W. (2024). Gut Microbial Community Structure and Carbon Source Metabolism Function of Adult *Picromerus lewisi*. Acta Entomol. Sin..

[35] Yang Y., Zhou Z., Qian X., Liu Y., Zhang M., Jiang M. (2024). Effects of Four Host Plants on Midgut Bacteria of Tuta absoluta. Chin. J. Biol. Control.

[36] Quast C., Pruesse E., Yilmaz P., Gerken J., Schweer T., Yarza P., Peplies J., Glöckner F.O. (2013). The SILVA ribosomal RNA gene database project: Improved data processing and web-based tools. Nucleic Acids Res..

[37] Wang Q., Garrity G.M., Tiedje J.M., Cole J.R. (2007). Naive Bayesian classifier for rapid assignment of rRNA sequences into the new bacterial taxonomy. Appl. Environ. Microbiol..

[38] Lozupone C., Lladser M.E., Knights D., Stombaugh J., Knight R. (2011). UniFrac: An effective distance metric for microbial community comparison. ISME J..

[39] Segata N., Izard J., Waldron L., Gevers D., Miropolsky L., Garrett W.S., Huttenhower C. (2011). Metagenomic biomarker discovery and explanation. Genome Biol..

[40] Ijaz M.U., Ahmed M.I., Zou X., Hussain M., Zhang M., Zhao F., Xu X., Zhou G., Li C. (2018). Beef, casein, and soy proteins differentially affect lipid metabolism, triglycerides accumulation and gut microbiota of high-fat diet-fed C57BL/6J mice. Front. Microbiol..

[41] Tang X.F., Sun Y.F., Liang Y.S., Yang K.Y., Chen P.T., Li H.S., Huang Y.H., Pang H. (2024). Metabolism, digestion, and horizontal transfer: Potential roles and interaction of symbiotic bacteria in the ladybird beetle *Novius pumilus* and their prey *Icerya aegyptiaca*. Microbiol. Spectr..

[42] Gu J., Yao Z., Lemaitre B., Cai Z., Zhang H., Li X. (2024). Intestinal commensal bacteria promote *Bactrocera dorsalis* larval development through the vitamin B6 synthesis pathway. Microbiome.

[43] Haider K., Abbas D., Galian J., Ghafar M.A., Kabir K., Ijaz M., Hussain M., Khan K.A., Ghramh H.A., Raza A. (2025). The multifaceted roles of gut microbiota in insect physiology, metabolism, and environmental adaptation: Implications for pest management strategies. World J. Microbiol. Biotechnol..

